# Diagnosis of membranous nephropathy with Anti-GBM glomerulonephritis: a case series report

**DOI:** 10.1186/s12882-024-03637-4

**Published:** 2024-06-21

**Authors:** Ge Liu, Xinyan Feng, Shuangyan Yu, Jie Sheng, Chunyan Liu, Lihua Wang

**Affiliations:** Department of Nephrology, The Second Hospital, Dalian Medical University, 467 Zhongshan RoadLiao Ning, Dalian, 116000 China

**Keywords:** Membranous nephropathy, Anti-glomerular basement membrane disease, Rapidly progressive glomerulonephritis

## Abstract

**Background:**

The concomitant occurrence of membranous nephropathy and anti-glomerular basement (anti-GBM) disease has been previously described but is extremely rare. However, delayed recognition or misdiagnosis leads to delayed treatment, resulting in worse renal and patient outcomes.

**Case presentation:**

We present 3 patients with rapidly progressive glomerulonephritis (RPGN), anti-GBM and serum-positive M-type phospholipase A2 receptor (anti-PLA2R) antibody. Renal biopsies revealed PLA2R-associated membranous nephropathy with anti-GBM glomerulonephritis. We analyzed the clinical and pathological characteristics and discussed that the correct diagnosis of membranous nephropathy with anti-GBM should rely on a combination of renal biopsy findings and serological testing. Despite aggressive treatment, one patient received maintenance hemodialysis, one patient progressed to CKD 3 stage, and the other patient died of cerebral infarction.

**Conclusion:**

The simultaneous occurrence of membranous nephropathy and anti-GBM disease is extremely rare. The correct diagnosis of membranous nephropathy with anti-GBM relies on a combination of renal biopsy findings and serological testing. Early diagnosis is needed to improve the renal dysfunction.

## Background

Membranous nephropathy (MN) is a common cause of nephrotic syndrome in adults, and it is characterized by the presence of subepithelial immune complexes followed by complement activation, basement membrane damage, and proteinuria. Fibrinoid necrosis and crescent formation are rarely encountered with MN. MN with crescents may occur as a “dual glomerulopathy” with superimposed antineutrophil cytoplasmic antibody (ANCA)-associated crescentic glomerulonephritis or, less often, anti-glomerular basement membrane (anti-GBM) antibodies. The combination of MN and anti-GBM disease has been well documented since the first report in 1974 [1]. This dual glomerulopathy likely represents the coincidental occurrence of two separate disease processes, MN followed by anti-GBM disease or the simultaneous presence of the two diseases [2–8]. Occasionally, anti-GBM followed by MN has also been reported in individual cases [9–12].

We report 3 cases on MN with anti-GBM disease and analyzed their clinical and pathological characteristics. The correct diagnosis of MN with anti-GBM disease should rely on a combination of renal biopsy findings and serological testing.

## Case presentation

### Case 1

A 67-year-old woman with severe edema and anuria for 14 days was admitted to our hospital. On admission, she had pitting edema in her lower legs, shifting dullness in her abdomen, which is a sign of ascites, and body weight gained of 2–5 kg. She had an upper respiratory tract infection and hemoptysis at home. Her body temperature was 36.4 °C, her pulse was 80 breaths/minute, and her blood pressure was 164/87 mmHg. Urinalysis revealed 4 + protein with numerous red blood cells in the sediment. Her hemoglobin level was 89 g/L, and her white blood cell count was 15.89 × 10^9^/L, with 87.7% neutrophils, 5.8% lymphocytes, 6.3% monocytes, 0% eosinophils, and 0.2% basophils. Her platelet count was 291 × 10^9^/L. Serum albumin level was 25 g/L. Renal function deteriorated rapidly, and her serum creatinine level was 7.79 mg/dL (normal range 0.7–1.4 mg/dL). Anti-GBM was positive, and the titer was > 200 RU/ml (negative range, less than 9 units). Serum M-type phospholipase A2 receptor antibody (anti-PLA2R) was positive (1:10 positive, indirect immunofluorescence assay). Her C-reactive protein level was 265.02 µg/dl, and her anti-streptolysin-O and anti-streptokinase titers were within normal ranges. Her serum cholesterol was slightly elevated (5.96 mmol/L). Antineutrophil cytoplasmic antibodies (ANCAs) were negative. Tests for anti-nuclear antibody (ANA), double-strand DNA (ds-DNA), extractable nuclear antibody (ENA), anti-SSA, anti-SSB, hepatitis B or C and human immunodeficiency virus were negative. Immunoglobulins (IgG, IgA, and IgM) and C3 and C4 levels were normal. Computed tomography (CT) of the chest revealed lung congestion and exudation. Renal ultrasound revealed that the sizes of both kidneys were normal (right, 10.5 × 4.9 cm; left, 11.4 × 4.7 cm), and the thickness of the cortex in both kidneys was normal (0.8 cm).

**Fig. 1:**
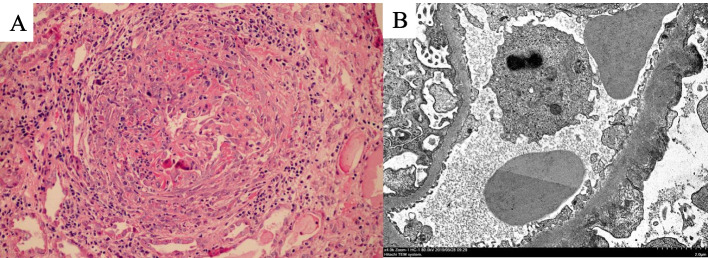
**A** Circumferential cellular crescent formation accompanying fibrin deposits (HE × 200). **B** Transmission electron microscopy image showing diffuse foot process effacement of the podocytes and subepithelial immunocomplex deposits (× 4000)

**Fig. 2:**
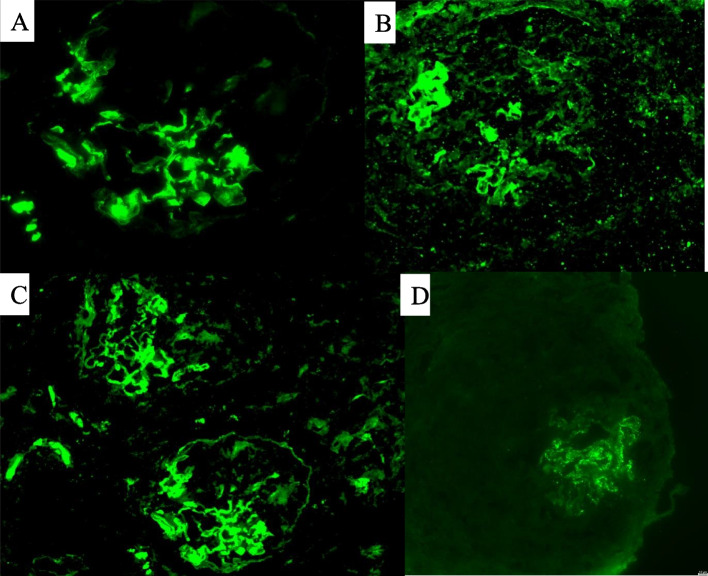
**A** Direct immunofluorescence reaction with FITC (fluorescein isothiocyanate)-labeled anti-IgG (note the simultaneous granular and linear staining). **B** Indirect immunofluorescence reaction with FITC-labeled anti-IgG3 (linear and granular staining). **C** Indirect immunofluorescence reaction with FITC-labeled anti-IgG4 (granular staining). **D** Indirect immunofluorescence reaction with FITC-labeled anti-PLA2R (granular staining)

Renal biopsy was performed for diagnosis. Light microscopy revealed crescentic glomerulonephritis with 15 of 17 glomeruli showing circumferential cellular crescent formation with fibrinoid necrosis. The glomerular capillary walls had spikes on PAM staining. Diffuse interstitial cell infiltration was observed with moderate tubular degeneration and interstitial fibrosis. Light microscopy pathology indicated the presence of crescentic glomerulonephritis (Fig. 1). Immunofluorescence demonstrated coexistent finely granular and linear depositions of IgG, IgG subclass except IgG4, C3 and κ and λ light chains along the glomerular capillary walls. However, PLA2R and IgG4 staining was granular along the glomerular capillary walls (Fig. 2). Electron microscopy revealed irregularly thickened glomerular basement membranes, rupture of the GBM and subepithelial electron-dense deposits. Podocytes revealed diffuse foot-process effacement and microvillous transformation (Fig. 1).

Based on these histological findings, MN stage II with anti-GBM glomerulonephritis was diagnosed. The patient received 10 plasma exchange treatments and a pulse of 500 mg intravenous methylprednisolone for 3 days during the early phase of the disease, followed by an oral prednisolone taper regimen (60 mg/day). After this aggressive treatment, renal function never improved, and the serum creatinine decreased to 4.52 mg/dL. The patient received maintenance hemodialysis therapy during follow-up.

### Case 2

A 70-year-old woman had a high fever with nausea and vomiting for 2 days. She had no history of arthralgia or skin rashes. On admission, swelling of the lower extremities was noted. Her body temperature was 36.2 °C, her pulse was 85 breaths/minute, and her blood pressure was 160/80 mmHg. Urinalysis revealed 3 + for protein with gross hematuria in the sediment. Her hemoglobin level was 74 g/L, and her white blood cell count was 23.03 × 10^9^/L, with 85.2% neutrophils, 8.3% lymphocytes, 6.3% monocytes, 0.1% eosinophils, and 0.1% basophils. Her platelet count was 75 × 10^9^/L. Serum albumin level was 23 g/L. Serum creatinine level was 6.33 mg/dL (normal range 0.7–1.4 mg/dL). Anti-GBM was positive, and the titer was 165.2 RU/ml (negative range, less than 9 units). Anti-PLA2R was positive (1:32 positive, indirect immunofluorescence assay). Her C-reactive protein level was 44.59 µg/dl, and titers of anti-streptolysin-O and anti-streptokinase were within normal ranges. She had no underlying disease. Renal ultrasound revealed that the sizes of both kidneys were normal (right, 9.7 × 4.3 cm; left, 9.8 × 4.5 cm), and the thickness of the cortex in both kidneys was normal (0.8 cm).

**Fig. 3:**
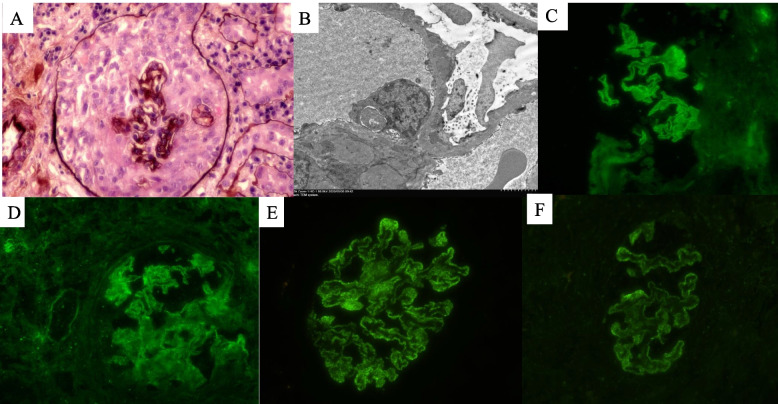
**A** Circumferential cellular crescent (PASM + HE × 400). **B** Transmission electron microscopy image of subepithelial immunocomplex deposits (× 2500). **C** Immunofluorescence (IF) staining showing global linear and granular deposition of IgG on capillary loops. **D**-**E** IgG subclass analysis showing positive linear and granular staining of IgG1 (**D**) and strong granular staining of IgG4 (**E**) on capillary loops. **F** IF staining showing global granular staining of PLA2R on capillary loops

Renal biopsy was performed for diagnosis. Light microscopy revealed exudative crescentic glomerulonephritis with cellular to fibro-cellular crescents involving 20 of 26 glomeruli, 6 of which exhibited fibrinoid necrosis. The glomerular capillary walls had spikes and bubbling on PAM staining. Light microscopy pathology indicated the presence of crescentic glomerulonephritis. The immunofluorescence portion of the specimen was near the obliteration of the glomerular tufts. The remnant glomerular basement membranes exhibited intense linear staining for IgG, IgG subclass excluded IgG4 and C3 along the glomerular capillary walls. The κ and λ light chains exhibited the same pattern. PLA2R and IgG4 staining was granular along the glomerular capillary walls. Electron microscopy revealed subepithelial electron-dense deposits with a distribution characteristic of membranous nephropathy involving all examined loops. Podocytes revealed diffuse foot-process effacement and microvillous transformation (Fig. 3). Electron microscopy revealed that the biopsy specimens were stage II MN.

Based on these histological findings, the patient was diagnosed as MN stage II with anti-GBM glomerulonephritis. The patient received 5 plasma exchange treatments and pulse intravenous methylprednisolone 240 mg for 3 days, followed by an oral prednisolone taper regimen (50 mg/day). Her serum creatinine decreased to 2.84 mg/dL. The patient developed chronic kidney disease (CKD) stage 3 without hemodialysis.

### Case 3

The patient was a 53-year-old woman who experienced nausea and vomiting for one week. She had a high fever with no history of underlying disease. On admission, her body temperature was 36.9 °C, her pulse was 76 beats/minute, and her blood pressure was 177/82 mmHg. Urinalysis revealed 4 + for protein with microscopic hematuria in the sediment. Her hemoglobin level was 84 g/L, and her white blood cell count was 14.22 × 10^9^/L, with 89.1% neutrophils, 8.3% lymphocytes, 2.4% monocytes, 0% eosinophils, and 0.2% basophils. Her platelet count was 162 × 10^9^/L. Her serum albumin level was 21 g/L. Her serum creatinine level was 13.57 mg/dL (normal range 0.7–1.4 mg/dL). Anti-GBM was positive, and the titer was > 200 RU/ml (negative range, less than 9 units). Anti-PLA2R was positive (1:32 positive, indirect immunofluorescence assay). C-reactive protein level was 93.67 µg/dl, and the titers of anti-streptolysin-O and anti-streptokinase were within normal ranges. She had no underlying disease. Renal ultrasound revealed that the sizes of both kidneys were normal (right, 10.1 × 5.4 cm; left, 10.5 × 4.7 cm), and the thickness of the cortex in both kidneys was normal (1.0 cm).

**Fig. 4:**
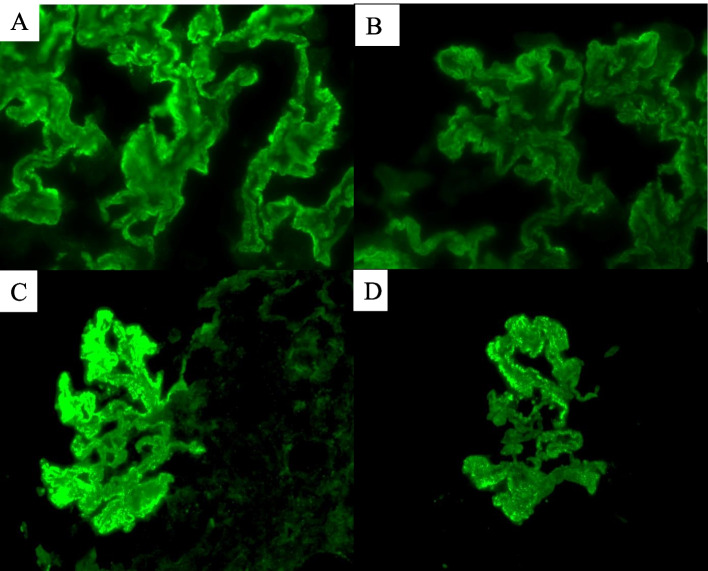
**A** IF staining showing global linear and granular deposition of IgG on capillary loops. **B** IF staining showing positive for IgG3 (global linear and granular deposition) on capillary loops. **C**-**D** IF staining showing strong granular deposition for IgG4 (**C**) and PLA2R (**D**) on capillary loops

Renal biopsy was performed for diagnosis. Light microscopy revealed crescentic glomerulonephritis with cellular crescents involving 24 of 31 glomeruli, 22 of which had fibrinoid necrosis. Light microscopy pathology indicated the presence of crescentic glomerulonephritis as case 1 and case 2. Immunofluorescence demonstrated the coexistence of fine granular and linear depositions of IgG, IgG subclass except IgG4, C3 and κ and λ light chains along the glomerular capillary walls. PLA2R and IgG4 staining was granular along the glomerular capillary walls (Fig. 4). Electron microscopy revealed diffuse podocyte foot-process effacement and microvillous transformation in biopsy specimens. Very few mesangial electron-dense deposits were observed, and subendothelial deposits were absent. There was no evidence of an organized substructure within the deposits, and tubulovesicular inclusions were not present.

Based on these histological findings, we diagnosed stage II MN with anti-GBM glomerulonephritis. After diagnosis, the patient received 3 plasma exchange treatments and continuous renal replacement treatment. Pulse intravenous methylprednisolone (500 mg) was administered for 3 days during the early phase of the disease, followed by an oral prednisolone taper regimen (60 mg/day). However, renal function never improved, and serum creatinine decreased to 10.18 mg/dL. The patient died of cerebral infarction one week after renal biopsy.

Table 1, Table 2 and Table 3 list the clinical and pathological features of the 3 patients.

**Table 1:** Clinical features of anti-GBM patients with MN

Variable	Case 1	Case 2	Case 3
Age and gender	67 F	70 F	53 F
Co-morbidity	None	None	None
Smoking status	Non-smoker	Non-smoker	Non-smoker
Hydrocarbon exposure	None	None	None
Prodromal infection	Yes	Yes	Yes
Presenting symptoms	Lethargy, dyspnea, edema, gross hematuria	Lethargy, dyspnea, edema, gross hematuria	Edema, microscopic hematuria
Renal status	AKI-RRT	AKI-RRT	AKI-RRT
Hemoptysis	Yes	No	Yes
C-reactive protein ug/dl	265.02	44.59	93.67
Hemoglobin (g/L)	89	74	84
WBC (× 10^9^/L)	15.89	23.03	14.22
Neutrophil (%)	87.7	85.2	89.1
Platelets (× 10^9^/L)	291	75	120
Creatinine (mg/dL)	7.79	6.33	13.57
Albumin (g/dL)	2.5	2.3	2.1
Anti-GBM titer (IU/ml; normal < 6.9)	> 200	165.2	> 200
ANCA	Negative	Negative	Negative
PLA2R	Positive	Positive	Positive
Treatment	Plasma exchange, cyclophosphamide, corticosteroids	Plasma exchange, cyclophosphamide, corticosteroids	Plasma exchange, cyclophosphamide, corticosteroids
Follow-up (days)	Receiving OP hemodialysis	Recovered kidney function, CKD 3 stage	Died of cerebral infarction
Last creatinine (mg/dL)	4.52	2.84	10.18

*Abbreviations: GBM* Glomerular basement membrane, *MN* Membranous nephropathy, *AKI* Acute kidney injury, *AKI-RRT* Acute kidney injury requiring renal replacement therapy, *WBC* White blood cell, *ANCA* Anti-neutrophil cytoplasm antibody, *PLA2R* M-type phospholipase A2 receptor, *CKD* Chronic kidney disease, *OP* Outpatient

**Table 2:** Pathological features of anti-GBM patients with MN

Variable	Case 1	Case 2	Case 3
MN stage	II	II	III
Glomerular lesions			
Sclerosis (% of total G)	0	7.7 (2/26 G)	6.5 (2/31 G)
Mild mesangial proliferation (% of total G)	0	15.4 (4/26 G)	16.1 (5/31 G)
Crescent components			
Cellular crescents (% of total G)	88.2 (15/17 G)	76.9 (20/26 G)	77.4 (24/31 G)
Circumferential cellular crescent	58.8 (10/17 G)	23.1 (6/26 G)	70.9 (22/31 G)
Fibrous-cellular crescents (% of total G)	29.4 (5/17 G)	3.8 (1/26 G)	0
Fibrous crescents (% of total G)	0	0	0
Tubular-interstitial damage ^a^			
Tubular atrophy	2	1	1
Interstitial fibrosis	2	1	2
Interstitial monocyte and lymphocyte infiltration	2	2	2

*Abbreviations: G* Glomeruli, *GBM* Glomerular basement membrane, *MN* Membranous nephropathy

^a^Semi-quantitative scoring of the tubulointerstitial lesions according to the percentage of the area involved: score 0 as 0% of the cortical area or tubules; score 1 as 25% of the cortical area or tubules; score 2 as 25–50% of the cortical area or tubules; and score 3 as > 50% of the cortical area or tubules

**Table 3:** IgG subclass and PLA2R distribution in anti-GBM patients with MN

Variable	Case 1	Case 2	Case 3	Distribution	Characteristics
IgG	4 +	3 +	3 +	Global	Linear and granular
IgG1	2 +	3 +	1 +	Global	Linear and granular
IgG2	1 +	1 +	1 +	Global	Linear and granular
IgG3	4 +	-	3 +	Global	Linear and granular
IgG4	4 +	4 +	4 +	Global	Fine granular
PLA2R	3 +	3 +	3 +	Global	Fine granular

*Abbreviations: IgG* Immunoglobulin G, *PLA2R* M-type phospholipase A2 receptor

## Discussion and conclusion

Fibrinoid necrosis and crescent formation are rarely encountered in the setting of MN. Crescents may be encountered in patients with MN lacking anti-GBM, ANCA, or clinical manifestations of lupus or chronic infections. In one report of MN with crescents [13], all patients were ANCA- or anti-GBM-negative. They found that 38% of biopsy specimens showed positive staining for PLA2R. These authors concluded that primary MN can also show crescents, perhaps due to severe glomerular damage related to immune complex deposition. These cases are limited to very few reports in the literature. In rare cases, MN with crescents may occur as a “dual glomerulopathy” with superimposed ANCA-associated crescentic glomerulonephritis or, less often, anti-GBM antibodies. This dual glomerulopathy likely represents the coincidental occurrence of two separate disease processes. From the perspective of ANCA-associated necrotizing and crescentic glomerulonephritis [14], the finding of coincidental MN may be associated with a greater degree of proteinuria and has a negative impact on the already poor prognosis of this condition. Therefore, the finding of glomerular fibrinoid necrosis or crescent formation in the setting of MN should prompt testing for ANCA. The association of anti-GBM glomerulonephritis with MN is very rare. Individual case reports and small series have documented the association of anti-GBM disease and MN presenting simultaneously or presenting sequentially with initial MN or initial anti-GBM disease [15].

The present report presented 3 cases of rapidly progressive glomerulonephritis, positive anti-GBM antibodies in the serum, and linear immunofluorescence staining for IgG and IgG subclass on renal biopsy specimens. Serum anti-PLA2R antibodies and granular immunofluorescence staining revealed MN. As shown in Table 1, the general symptoms of our 3 patients were a rapid decline in kidney function, nephrotic range proteinuria, and hypoalbuminemia. Pulmonary involvement with hemoptysis in the form of Goodpasture syndrome has also been described. All 3 patients had previous prodromal infections, and 2 patients had hemoptysis. The discovery of concomitant disease was accidental in our 3 patients. The rapid progression of the disease suggested screening for anti-GBM antibodies. However, our patients also had nephrotic-range proteinuria and hypoalbuminemia, which led us to investigate the anti-PLA2R titer. All 3 patients were PLA2R antibody-positive. Immunofluorescent PLA2R antigen staining was granular, which indicated primary membranous nephropathy. Although the 3 patients had not been admitted to our hospital before, we diagnosed stage II MN with anti-GBM glomerulonephritis based on clinical and pathological data.

Zhao et al. [16] reported that patients with fever presented with higher levels of serum anti-GBM antibodies and serum creatinine, a greater percentage of crescents, a greater incidence of oliguria/anuria and a greater percentage of ESRD. They concluded that infections may participate in the initiation and exacerbation of anti-GBM disease. Maria et al. reported greater incidences of anti-GBM disease and severe acute respiratory syndrome coronavirus 2 (SARS-CoV-2) infection [17]. Therefore, there is a potential for viral infection to trigger secondary autoimmunity, including the incidence of rapid-progressive glomerulonephritis. All 3 of our patients had previous prodromal infections. These infections may have triggered secondary autoimmunity that resulted in anti-GBM disease. Whether anti-GBM or anti-PLA2R-associated membranous nephropathy occurs first is unknown.

Zhao [18] et al. reported that natural anti-GBM autoantibodies with low titers and low quantities existed in normal human sera. Natural and disease-associated autoantibodies against GBM primarily differ in IgG subclass, antibody quantity and avidity. In anti-GBM disease, increased levels of autoantibodies may cause pathological events, which can occur due to infection, smoking, exposure to hydrocarbons, or degradation by reactive oxygen species. They found that the predominant IgG autoantibodies were IgG1 and IgG4 in anti-GBM disease patients, while the predominant natural IgG autoantibodies to GBM were the IgG2 and IgG4 isotypes [18]. IgG4 has a poor ability to activate complement [19]. Notably, autoantibodies of the IgG1 and IgG3 subclass are known to activate complement. Once complement is activated, complement chemotactic factors may attract effector cells, such as neutrophils and macrophages, which play distinct roles in mediating anti-GBM glomerulonephritis.

Immunofluorescence revealed variably intense fine granular and linear staining for IgG, IgG subclass, and κ and λ light chains involving GBMs in our 3 patients. On high-power examination, staining was resolved as an inner layer of linear positivity and an outer layer of fine-to coarse granular staining. There was diffuse granular and linear staining of glomerular capillary walls for complement C3. Immunofluorescence staining for the PLA2R antigen and IgG4 revealed a strong granular pattern in 3 patients. We could not obtain clinical data before the 3 patients admitted to our hospital, so subepithelial electron-dense deposits were identified in a global distribution by electron microscopy, which indicated that MN preceded the anti-GBM disease.

However, there are no precise recommendations for therapy for MN combined with anti-GBM disease, and we used treatment according to the KDIGO recommendations for anti-GBM disease [20]. In addition to immunosuppressive therapy and plasmapheresis, the prognosis is primarily dependent on a rapid diagnosis. The longer the time from diagnosis, the greater the extent of global sclerosis and tubulointerstitial chronicity, and the worse the prognosis. Delayed recognition or misdiagnosis can lead to delayed treatment, which worsens renal and patient outcomes, and increases financial costs. Among our 3 patients, patient 2 experienced nausea and vomiting for 2 days, and we performed renal biopsy in the early phase. Light microscopy demonstrated fewer and smaller crescents and mild tubular atrophy and interstitial fibrosis. The patient received 5 plasma exchanges and pulse intravenous methylprednisolone (240 mg) for 3 days, followed by an oral prednisolone taper regimen. Her serum creatinine decreased to 2.84 mg/dL. However, patient 1 was admitted to our hospital 14 days after disease onset, and renal biopsy revealed a greater percentage of crescents, moderate tubular atrophy and interstitial fibrosis, which have a worse prognosis.

Progression to end-stage renal disease in these patients is not certain. There was an interesting review from Troxell [15] who analyzed the demographics of previously reported patients. They reported 5 cases of anti-GBM disease preceding MN in young adults, most of whom initially presented with hematuria or hemoptysis. MN was documented 5–28 months later. The outcome was generally favorable. The simultaneous presentation of anti-GBM disease and MN included 8 young adults and 9 middle-aged to elderly patients. The clinical presentations most commonly included hematuria or hemoptysis and may or may not have included edema or proteinuria. Renal function recovered in some patients (6/17), especially the young adults. The 5 patients who initially presented with MN followed by anti-GBM disease were middle-aged to elderly. Most patients in this group presented with edema consistent with MN. Progression to anti-GBM disease 9–20 months later was associated with hematuria, nausea/vomiting and renal failure. Our 3 patients were all elderly who had initial membranous nephropathy with subsequent anti-GBM disease. Despite aggressive treatment, one patient received maintenance hemodialysis, one patient progressed to CKD 3 stage, and the other patient died of cerebral infarction.

The correct diagnosis of MN with anti-GBM disease relied on a combination of renal biopsy findings and serological testing. Recognizing linear staining of IgG and IgG subclass in MN can be technically difficult because of the intense granular staining of immune complex deposits. A misdiagnosis of pauci-immune crescentic glomerulonephritis may be made because no glomerular linear staining is observed. Therefore, careful examination under a high-powered lens is needed. Careful examination of the background fluorescence must be performed before concluding the absence of glomerular linear staining. Linear immunofluorescence staining along the GBM may be observed in settings other than anti-GBM glomerulonephritis, such as diabetic nephropathy. In diabetic nephropathy, the linear accentuation of the GBM with anti-IgG is typically not accompanied by linear C3 staining, but nonspecific C3 staining may be observed. Tubular and Bowman’s capsular basement membranes may also show linear accentuation of the GBM with anti-IgG in diabetic nephropathy, but this staining is weaker than the GBM [21]. In anti-GBM antibody-mediated glomerulonephritis, variable granular or linear C3 staining is present, and the latter pattern may be continuous or discontinuous. The presence of linear C3 may help support a diagnosis of anti-GBM-related disease.

Anti-GBM disease combined with another glomerular disease results in rapid deterioration of renal function and leads to end-stage renal disease. Therefore, early recognition is the important method for preserving kidney function. Regardless of the underlying mechanism, our case series provides an important practical message for clinicians managing patients with anti-GBM disease. Smoking and hydrocarbon exposure should be forbidden once the diagnosis is suspected to avoid precipitation of a life-threatening pulmonary hemorrhage. Infection is a common clinical symptom of anti-GBM disease and is associated with severe kidney injury, and it should be avoided. Careful examination of the background fluorescence must be performed before the final diagnosis.

We reported 3 patients with MN superimposed on anti-GBM disease. Our current series of patients with concurrent MN and anti-GBM disease confirmed the association of these 2 diseases and adds clinical and histopathological data to the literature. Although the pathophysiology of the combined disease is not well understood, the simultaneous appearance of disease in our patients suggested the possibility of the release of GBM antigens with in situ immune complex formation and/or epitope exposure as causes of the combined disease spectrum. Early diagnosis is needed to improve renal dysfunction.

## Data Availability

The datasets related to this case report are available from the corresponding author.

## Abbreviations

G: Glomeruli
GBM: Glomerular basement membrane
MN: Membranous nephropathy
AKI: Acute kidney injury
AKI-RRT: Acute kidney injury requiring renal replacement therapy
WBC: White blood cell
ANCA: Anti-neutrophil cytoplasm antibody
PLA2R: M-type phospholipase A2 receptor
IgG: Immunoglobulins G
IgA: Immunoglobulins A
IgM: Immunoglobulins M
CKD: Chronic kidney disease
OP: Outpatient
